# Supplementary figures and images for: Genome-Wide Association Analysis Identified Variants Associated with Body Measurement and Reproduction Traits in Shaziling Pigs
Source: Genes (Basel). 2023 Feb 18;14(2):522. doi: 10.3390/genes14020522 (PMC9957351; doi:10.3390/genes14020522)

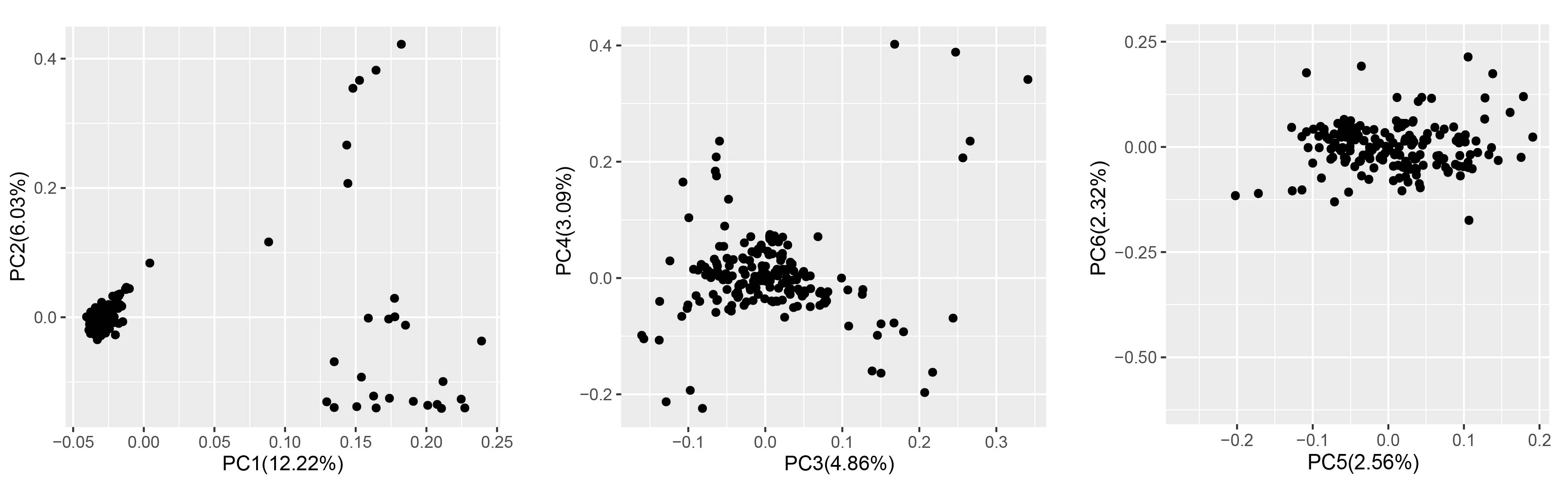

Supplement: Supplementary file 1 [file genes-14-00522-s001.zip › Figure. S1.jpg]

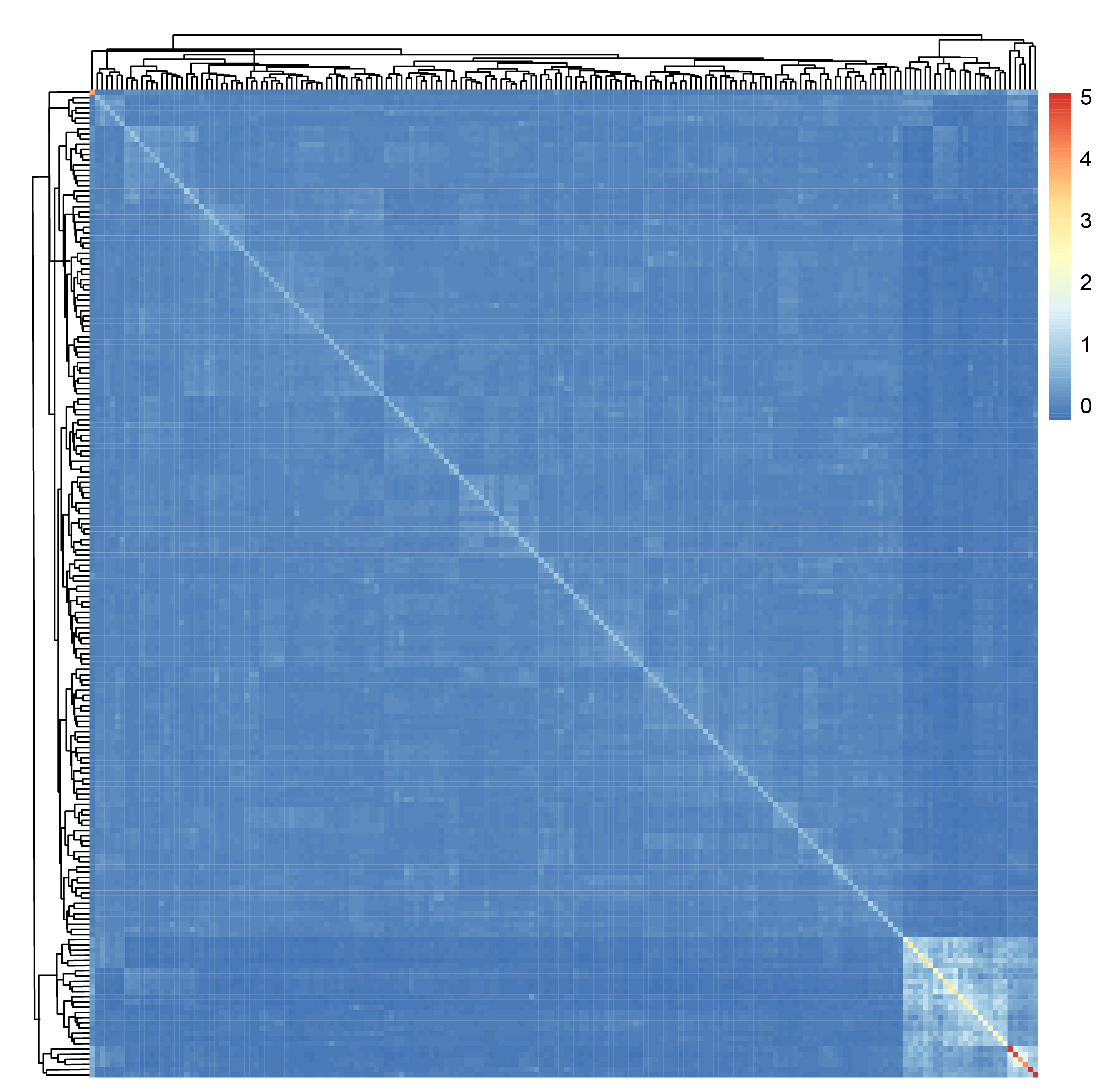

Supplement: Supplementary file 1 [file genes-14-00522-s001.zip › Figure. S2.jpg]

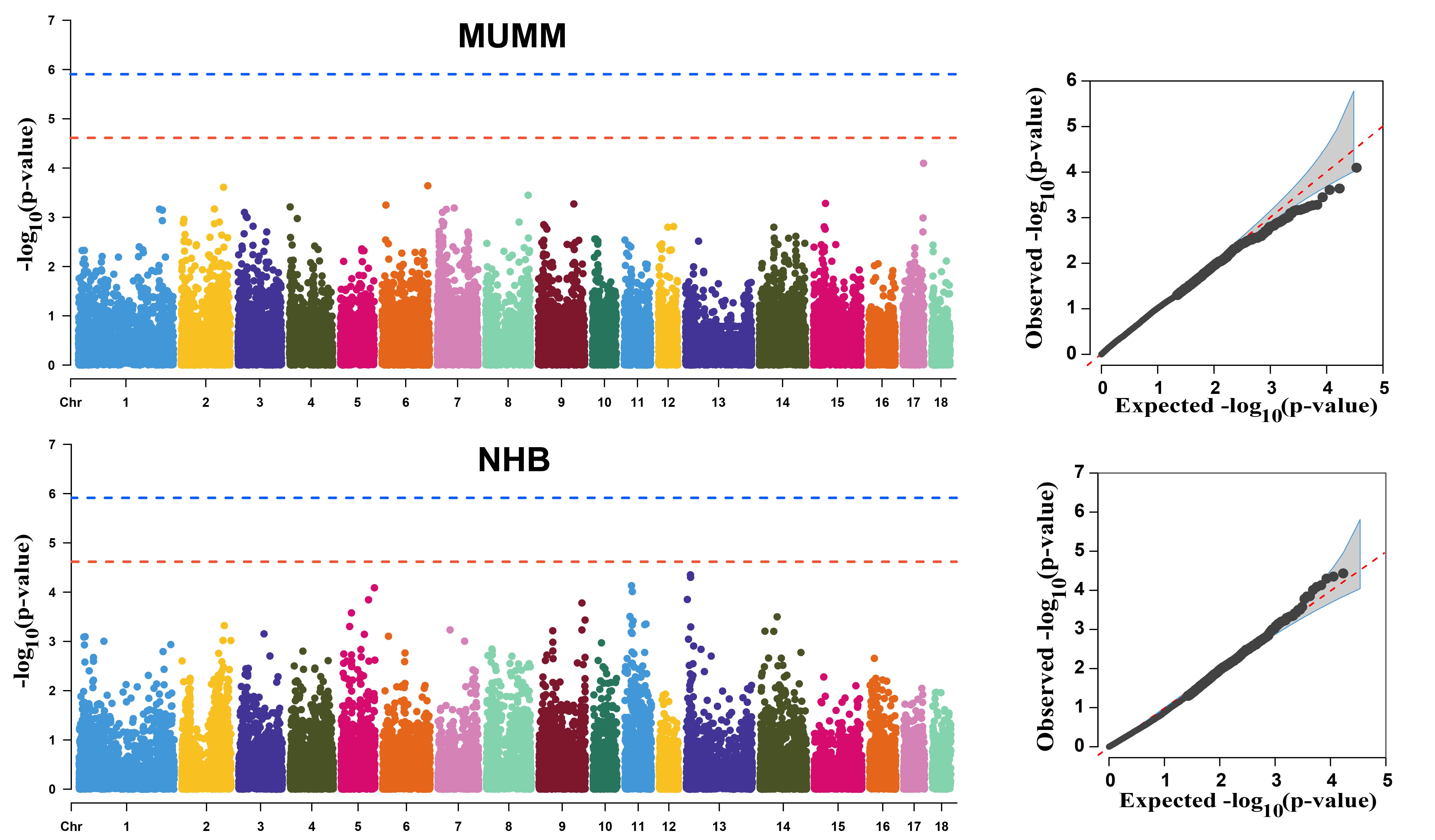

Supplement: Supplementary file 1 [file genes-14-00522-s001.zip › Figure. S3.jpg]
